# Circular RNAs: emerging players in asthma and COPD

**DOI:** 10.3389/fcell.2023.1267792

**Published:** 2023-11-24

**Authors:** Xiaoying Liu, Md Khadem Ali, Kamal Dua, Yuqiang Mao, Jun Liu

**Affiliations:** ^1^ Department of Breast Surgery, The First Affiliated Hospital of China Medical University, Shenyang, China; ^2^ Pre-Professional Health Academic Program, California State University, Hayward, CA, United States; ^3^ Discipline of Pharmacy, Graduate School of Health, University of Technology Sydney, Sydney, NSW, Australia; ^4^ Faculty of Health, Australian Research Centre in Complementary and Integrative Medicine, University of Technology Sydney, Ultimo, NSW, Australia; ^5^ Uttaranchal Institute of Pharmaceutical Sciences, Uttaranchal University, Dehradun, India; ^6^ Department of Thoracic Surgery, Shengjing Hospital of China Medical University, Shenyang, China; ^7^ Clinical Skills Practice Teaching Center, Shengjing Hospital of China Medical University, Shenyang, China

**Keywords:** CircRNAs, miRNAs, lung disease, asthma, COPD

## Abstract

Circular RNAs (circRNAs) belong to a unique class of endogenously expressed non-protein-coding RNAs with a distinct circularized structure, characterized by the absence of 5′-cap and 3′-polyadenylate ends. They are generally formed through back-splicing from pre-mRNAs. They serve as regulators of transcription and splicing, and act as sponges for microRNAs (miRNAs) and RNA-binding proteins, thereby modulating the expression of target genes. As a result, they exert a substantial impact on a diverse array of cellular and biological processes, including cell proliferation, migration, inflammation, and oxidative stress. Asthma and COPD are chronic airway conditions that currently have no cure. In recent years, emerging evidence suggests that altered expression of circRNAs in airway, bronchial and immune cells is involved in asthma and COPD pathogenesis. Studies exploring circRNA dysregulation in asthma have showcased their involvement in regulating the proliferation, migration, and inflammation of airway smooth muscle and bronchial epithelial cells, as well as impacting goblet cell metaplasia, Th2 cell differentiation, and macrophage activation, primarily through interactions with miRNAs. Similarly, in COPD, circRNAs have shown altered expression patterns in the blood and lungs of patients, and these changes have been linked to modulating inflammation, oxidative stress, and airway remodeling in preclinical models. Furthermore, certain circRNAs have demonstrated promising potential as diagnostic and prognostic biomarkers for both asthma and COPD. This review delves into the current understanding of the function and molecular mechanisms of circRNAs in asthma and COPD, along with exploring their potential as biomarkers in these respiratory conditions.

## Introduction

Chronic respiratory diseases (CRDs) represent a group of lung conditions that affects the respiratory system, particularly the airways and lungs. These diseases are characterized by reduced lung function, and, in some cases, irreversible damage to respiratory tissues. In 2019, CRDs ranked as the third leading cause of global mortality associated with a significant social and economic burden, thereby posing a significant healthcare challenge (Diseases and Injuries, 2020; Syamlal et al., 2020). The most common CRDs include asthma and chronic obstructive pulmonary disease (COPD).

Asthma is a multifaceted and heterogeneous airway condition, affecting an estimated 260 million people globally (Ali et al., 2020; Liu et al., 2022; The Global Asthma Report, 2022). Its cardinal clinical manifestations encompass airflow obstruction, breathing difficulties, coughing, chest tightness, and wheezing (Habib et al., 2022). While asthma can develop at any age, it is most common in children. The exact cause of asthma remains unknown; however, it is believed to arise from a combination of genetic factors (individuals with a family history of asthma) and environmental influences (such as allergies, air pollution, cold air, and exercise). These contributing factors influence the pathogenic changes within the airways, including inflammation, mucous hypersecretion, airway remodeling, airway hyper-responsiveness (AHR), and airway obstruction (Gysens et al., 2022). Airway remodeling involves significant structural alterations in the airways, which include hypertrophy/hyperplasia of airway smooth muscle cells (ASMCs), alterations in airway epithelial cells (AECs), sub-epithelial fibrosis, goblet cell metaplasia, and increased expression of angiogenic factors (Curran and Cohn, 2010). While available medications can effectively manage the symptoms, a cure for the disease is currently lacking. Therefore, there is a pressing need to develop an effective therapy that can target the underlying causes of the disease.

Chronic Obstructive Pulmonary Disease (COPD) comprises a spectrum of lung conditions, including pulmonary emphysema and chronic bronchitis, which lead to breathing difficulties and airflow obstruction (Guo et al., 2022). COPD is a major global contributor to mortality and morbidity, placing a substantial economic and social burden worldwide (Safiri et al., 2022). While COPD can affect individuals of all ages, it is more commonly observed in older populations (Tudorache et al., 2017; Matera et al., 2023). Although smokers are at a higher risk of developing COPD, non-smokers can also be affected by the disease due to exposure to environmental pollutants, occupational dust and chemicals, respiratory infections, and genetic factors (Feizi et al., 2022; He et al., 2023). Despite extensive efforts to identify effective therapies for COPD, the disease remains incurable, and the existing treatment options only aim to manage clinical symptoms, slow down disease progression, and enhance the quality of life (Bollmeier and Hartmann, 2020). Consequently, the development of efficient treatments for this debilitating condition is urgently needed.

Non-coding RNAs (ncRNAs) are a class of RNA molecules that do not encode proteins. NcRNAs play essential regulatory roles in various cellular and biological functions, including cellular proliferation, migration, apoptosis, angiogenesis, inflammation, and metabolism in both health and disease (Bhatti et al., 2021). Based on their size, ncRNAs can be categorized into different subtypes, which include microRNAs (miRNAs, approximately 21–25 nucleotides in length), long non-coding RNAs (lncRNAs, over 200 nucleotides), circular RNAs (circRNAs, roughly 100 nucleotides to 4 kilobases, but in a circularized form), and more. They are involved in the regulation of gene expression at various levels (Bhatti et al., 2021). NcRNAs have gained significant attention in recent years due to their roles in various diseases, including cancer, neurodegenerative disorders, and lung diseases (Bhatti et al., 2021). Their study has opened new avenues for understanding disease mechanisms and exploring potential therapeutic interventions. While miRNAs and lncRNAs have been extensively investigated in both healthy and diseased states, circRNAs have received comparatively less attention in research.

CircRNAs represent a distinct class of endogenously expressed circularized RNA molecules lacking 5′end caps and 3′poly(A) ends. Generally, circRNAs do not have the capacity to encode proteins. Nevertheless, recent studies have unveiled some exceptions, where certain circRNAs have been found to translate into proteins. An example of this is circ-FBXW7, which has been identified as a circRNA capable of translating into the protein FBXW7-185aa (Yang et al., 2018), although, its functions remain largely unknown. CircRNAs are characterized by their distinct covalently closed ring structure, mainly generated via head-to-tail splicing. This structure of circRNAs confers protection against degradation by RNA endonucleases, resulting in their remarkable stability and high abundance in blood and tissues (Wang et al., 2017; Li X. et al., 2018). Additionally, this structural characteristic helps them to be highly conserved across different species. Most circRNAs display prominent expression patterns that are specific to particular cells, tissues, and stages of development.

**FIGURE 1:**
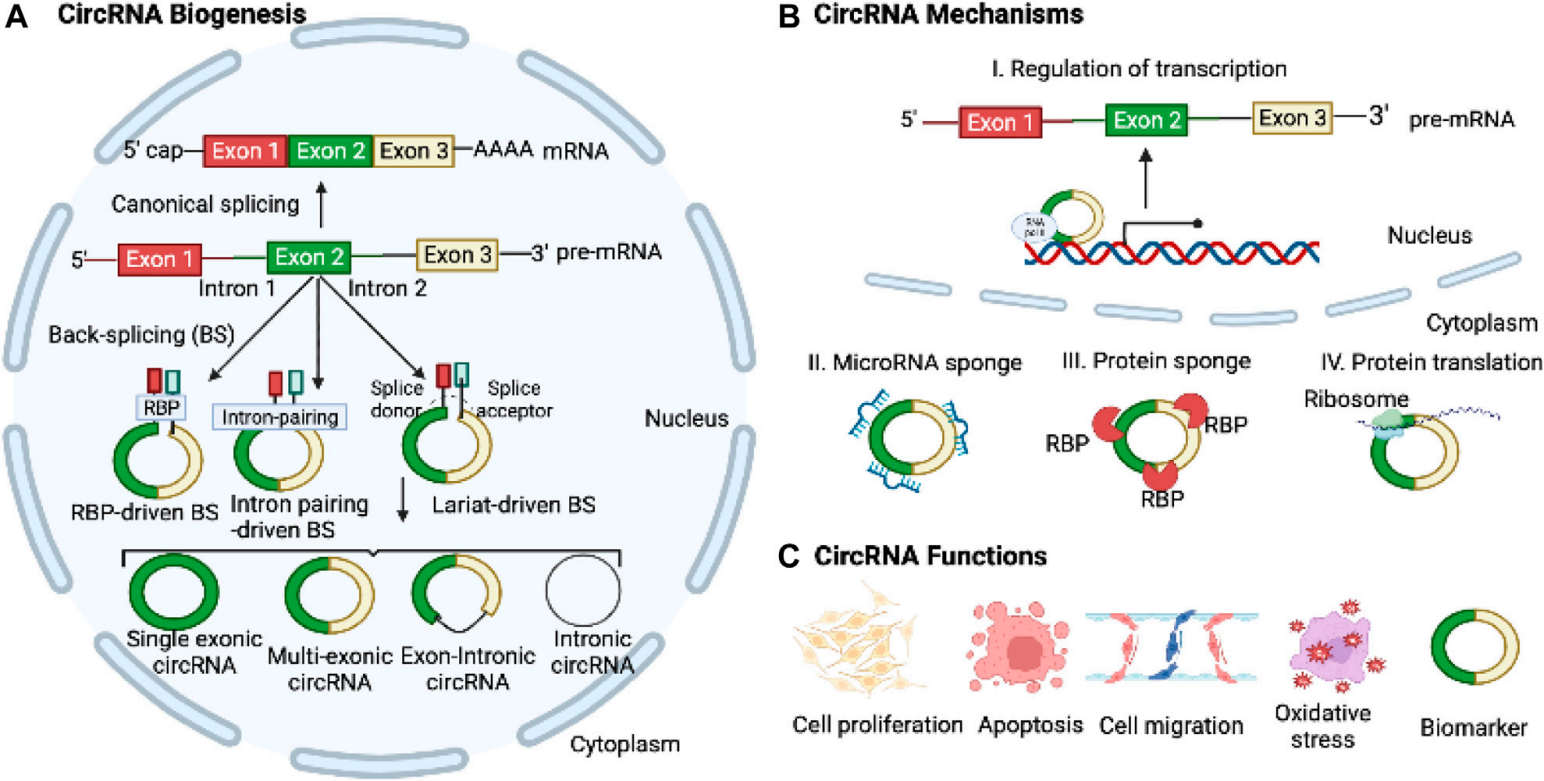
Biogenesis, Mechanisms, and Functions of CircRNAs. **(A)** CircRNAs are derived from pre-mRNAs through three widely accepted mechanisms: intron-pairing-driven, RBP-mediated, and lariat-driven back-splicing processes. **(B)** CircRNAs play regulatory roles in transcription and translation and act as microRNA sponges to control target gene expressions. **(C)** CircRNAs can modulate cell proliferation, apoptosis, migration, EMT, and oxidative stress. Moreover, they hold potential as diagnostic and prognostic biomarkers for various diseases.

Most circRNAs are generated through back-splicing, which involves the circularization of pre-mRNA derived from protein-coding genes, antisense transcripts, the intergenic spacer in tRNA, or lncRNAs (Ali et al., 2022). CircRNAs can originate from both exons and intron regions. Currently, circRNAs are categorized into four groups based on their synthesis mechanisms and the genomic splice junctions where they are formed: intronic, exonic, exon-intron, and tRNA intronic circRNAs, the latter being generated by tRNA introns. Exonic circRNAs, being the most prevalent type among circRNAs, are primarily located in the cytoplasm. In contrast, other types of circRNAs are mostly detected in the nucleus, suggesting their potential role in regulating gene transcription. There are three well-established models for circRNA formation: 1) Intron pairing-driven circularization, where inverted repeat elements like ALU repeats, facilitate splicing by bringing splice donor and acceptor sites together; 2) intron lariat generation and exon skipping and, where exonic circRNAs are formed by joining exon ends; and 3) RBP-associated mechanism, where RBPs bridge flanking introns to facilitate loop formation (Figure 1). The biogenesis of the circRNAs can be modulated by several factors, including the cis-elements (intronic complementary sequences), canonical spliceosomal machinery, and transfactors (RBPs) (Ali et al., 2022).

CircRNAs have emerged as key regulators in a diverse array of physiological, biological, and cellular processes, encompassing cellular proliferation, migration, and apoptosis through their interactions with other RNAs and proteins (Salzman, 2016; Li X. et al., 2018; Mao and Xu, 2020; Ali et al., 2022). MicroRNA sponging is a well-established mechanism of circRNAs, wherein they modulate the expression of target mRNA genes by competitively binding to specific microRNAs (Salzman, 2016). CircRNAs also bind to RBPs and other proteins, acting as translational regulators and serving as protein scaffolds to regulate target gene expression (Salzman, 2016). Recently, it has been discovered that circRNAs can also be translated into polypeptides (Zhou et al., 2020). The covalently closed structure of circRNAs gives them a longer half-life and greater resistance to RNase R compared to linear RNAs. Moreover, circRNAs exhibit differential expression patterns in various diseases, encompassing diverse conditions such as cancer and cardiovascular diseases (Altesha et al., 2019; Li et al., 2021; Kristensen et al., 2022). Given these features, circRNAs hold great promise as potential diagnostic and therapeutic biomarkers for a wide range of diseases.

**FIGURE 2:**
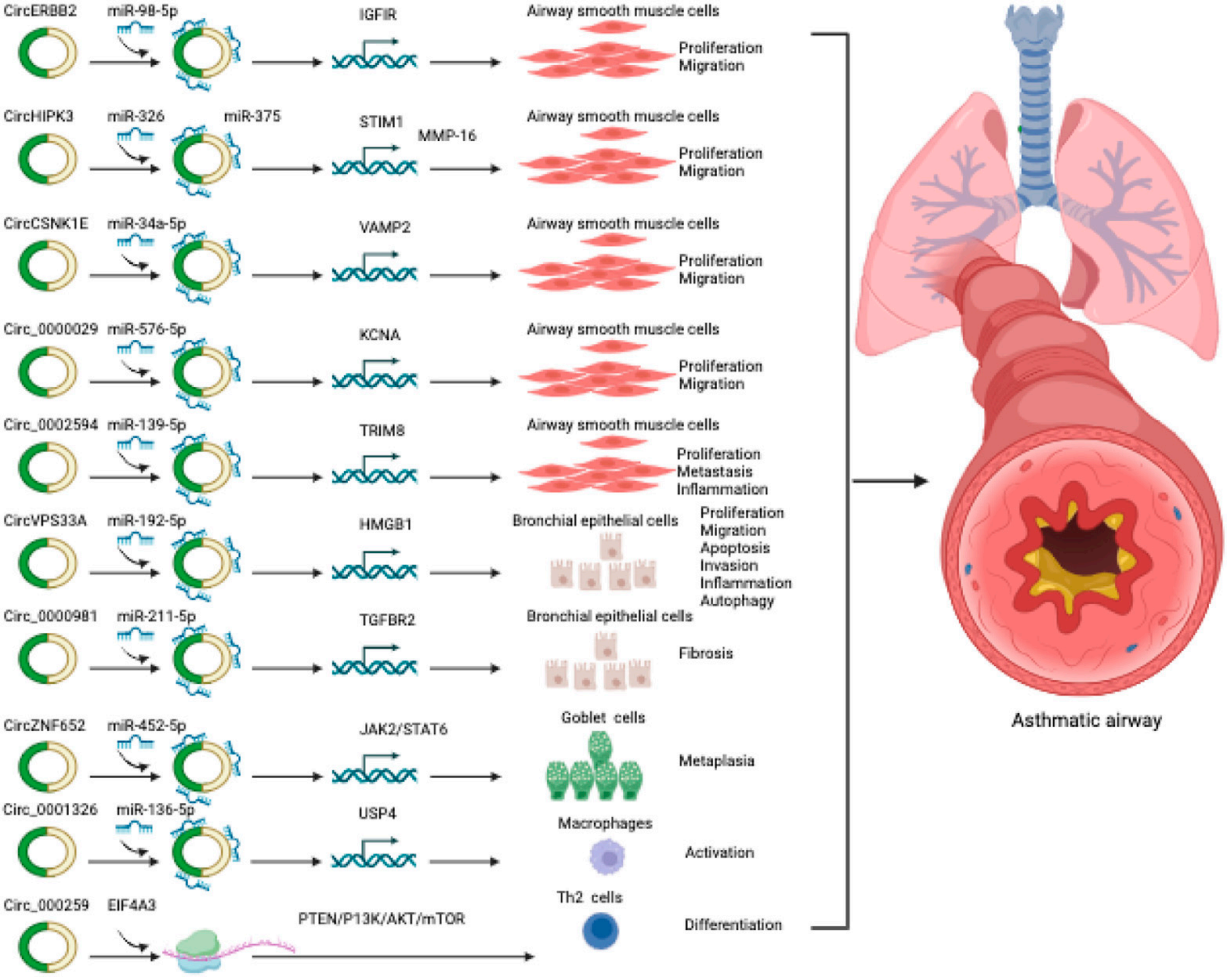
CircRNAs involved in asthma.

**FIGURE 3:**
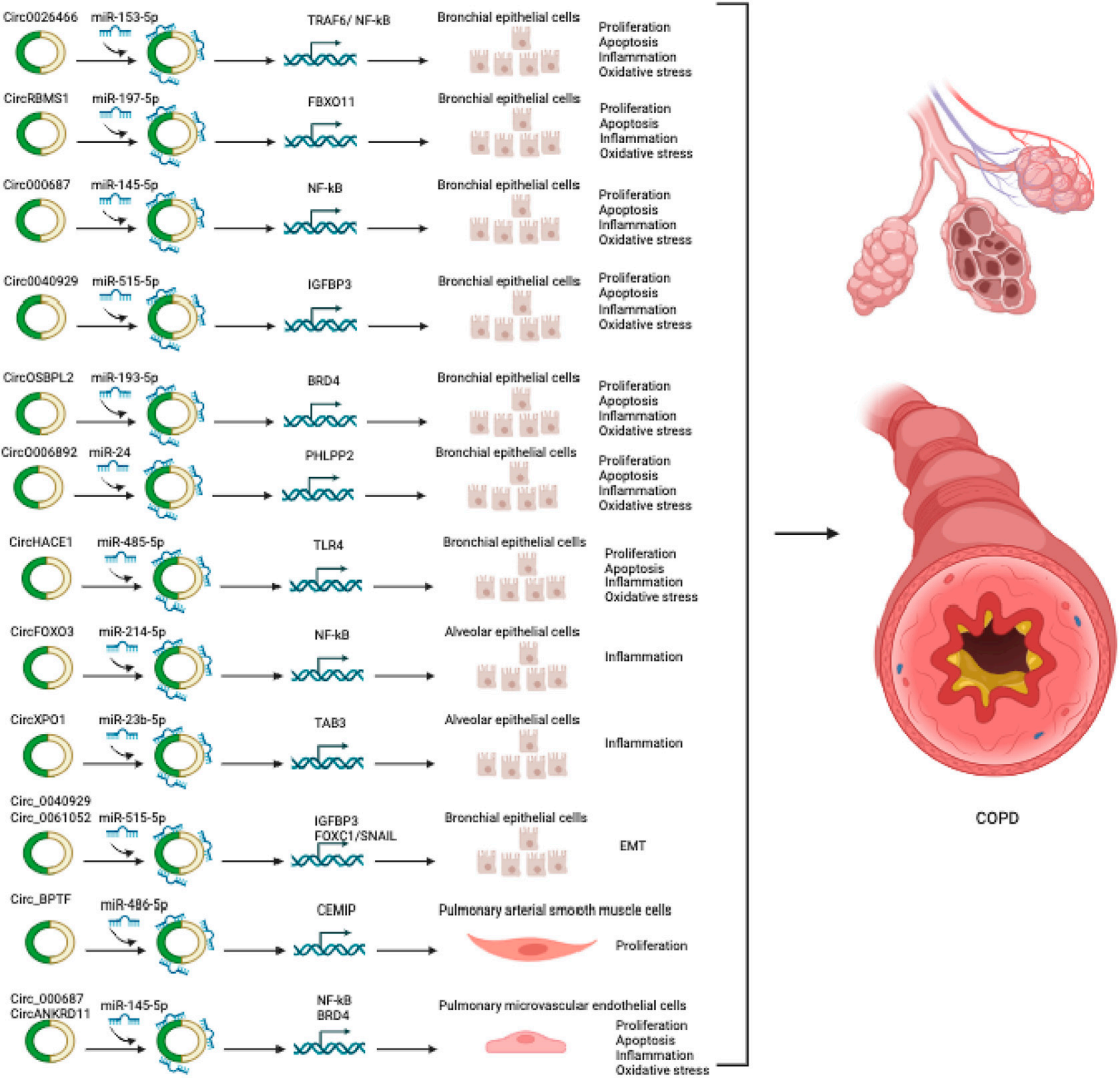
CircRNAs involved in COPD.

Recent findings indicate dysregulation of circRNAs in both the blood and lungs of patients with asthma and COPD (Tables 1–4). These circRNAs have demonstrated significant regulatory effects on the pathophysiology of these respiratory conditions (Figures 2, 3). This review presents a comprehensive overview of circRNAs and their relevance to the pathophysiology of asthma and COPD. We specifically concentrate on asthma and COPD among the various CRDs, as they are the most prevalent, and currently there is no comprehensive reviews solely regarding the involvement of circRNAs in these conditions. Our focus lies primarily on the function and mechanisms of circRNAs in the pathophysiology of these respiratory conditions. Moreover, we delve into the potential of circRNAs as biomarkers and therapeutic targets for asthma and COPD.

**TABLE 1:** Emerging role of circRNAs in the pathogenesis of asthma.

CircRNA	Expression level	Key role	Mechanisms	References
Circ_0002594	↑ in CD4^+^ T cells of asthmatics	Associated with SPT-positive and Th2-high biomarker profiles	-	Huang et al. (2021b)
	↑ in CD4^+^ T cells of asthmatics	Regulates Th2 cell differentiation	Binds to EIF4A3 to downregulate PTEN, activates PI3K/AKT/mTOR	Wang and Cheng (2022)
	↑ in asthmatics and PDGF-treated ASMCs	Knockdown could inhibit PDGF- induced ASMCs damage	Sponges with miR-139-5p to regulate TRIM8	Quan et al. (2022)
circERBB2	↑ in bronchial biopsy in asthmatics and PDGF-treated ASMCs	Knockdown attenuates proliferation and migration of ASMCs	circERBB2/miR-98-5p/IGF1R signaling axis	Huang et al. (2021a)
CircZNF652	↑ in BALF and airway epithelia of asthmatics, in airway epithelia in animal models of asthma	Accelerates goblet cell metaplasia	circZNF652/miR-452-5p/JAK2/STAT6 signaling axis	Wang et al. (2022a)
circSORT1	↑ in induced sputum cells of asthmatics	correlated with asthma clinical parameters, such as FeNO, EOS%, IL-17A, IFN-γ, and PC20	CircSORT1/has-miR-185-3p/ZNFX1	Xu et al. (2023)
circSERPINB1	↑in induced sputum cells of asthmatics	Correlated with asthma clinical parameters, such as IL-6, IL-17A, IFN-γ, FEV1%, and FVC%	-	Xu et al. (2023)
circHIPK3	↑ in ASMCs stimulated with PDGF-BB	Inhibition attenuated proliferation, migration, and apoptosis of ASMCs treated with PDGF	circHIPK3/miR-326/STIM1 axis	Lin et al. (2020)
	↑ in ASMCs stimulated with PDGF-BB	Silencing inhibited proliferation, migration, and invasion and promoted apoptosis of PDGF-treated AMSCs	circHIPK3/miR-375/MMP-16 axis	Jiang et al. (2021)
circ_406961	↓ in BEAS-2B exposed to PM2.5	suppressed PM_2.5_-induced inflammation	Circ_406961/STAT3/JNK/ILF2 axis	Jia et al. (2020)
circ_CSNK1E	↑ in PDGF-treated ASMCs	Knockdown inhibited PDGF-induced proliferation and migration of ASMCs	Circ_CSNK1E/miR-34a-5p/VAMP2 axis	Ding et al. (2022)
circVPS33A	↑ in plasma of asthmatics and in BEAS-2B cells treated with Der p1	Inhibition reduced cells injury	CircVPS33A/miR-192-5p/HMGB1 axis	Su et al. (2021)
circ_0005519	↑ in PBMCs and CD4^+^ T cells of asthmatics	Positively associated with exhaled NO and the peripheral blood eosinophil ratio	Circ_0005519/hsa-let-7a-5p//IL-13/IL-6 axis	Huang et al. (2019)
Circ_0000029	↓ in ASMCs treated with PDGF-BB	Regulates viability, apoptosis, and migration of ASMCs	Circ_0000029/miR-576-5p/KCNA	Wang et al. (2023a)
circ_0000981	↑ in the lungs of Ova-induced asthma models and TGFb-treated TC-1 cells	Promotes pulmonary fibrosis	Mmu_circ_0000981/miR-211-5p/TGFBR2 Axis	Zeng et al. (2021)
Circ 001372	↓ in serum of Ova-induced asthma mice models	Inhibits inflammation	Circ 001372/miRNA-128-3p/NFAT5 axis	Lin and Wan (2022)
Circ_0000629 Circ_0000455 Circ_0000909	↑ in the lung of HDM-induced asthma	-	Circ_0000455 and Circ_0000629 could target miR-29b and miR-15a	Bao et al. (2020)
Circ_0000723 Circ_0001389 Circ_0001454	↓ in the lung of HDM-induced asthma	-	Circ_0001454 and Circ_0000723 could target miR-146b and miR-214	Bao et al. (2020)

**TABLE 2:** Biomarker role of circRNAs in asthma.

CircRNAs	Expression level	Study size	Biomarker role	Biomarker type	Refs
Circ_0002594	↑ in CD4^+^ T cells of asthmatics	Healthy *n* = 54	High AUC values in the presence of ICS or not	Diagnostic Prognostic	Huang et al. (2021b)
		Asthmatics -ICS *n* = 83			
		Asthmatics + ICS *n* = 48			
CircSORT1	↑ induced sputum of asthmatics	Healthy *n* = 20	Higher specificity and sensitivity in predicting asthma, and correlated with clinical asthma parameters, such as FeNO, EOS%, IL-17A, IFN-γ, and PC20	Diagnostic Prognostic	Xu et al. (2023)
		Asthmatics *n* = 68			
CircSERPINB1	↑ induced sputum of asthmatics	Healthy *n* = 20	Higher specificity and sensitivity in predicting asthma, and correlated with clinical asthma parameters, such as IL-6, IL-17A, IFN-γ, FEV1%, and FVC%	Diagnostic Prognostic	Xu et al. (2023)
		Asthmatics *n* = 68			
Circ_0005519	↑ in CD4^+^ T cells and PBMCs of asthmatics	Healthy *n* = 24 (CD4^+^ T)	Correlates with exhaled nitric oxide	Prognostic	Huang et al. (2019)
		Asthmatics *n* = 30 (CD4^+^ T)			
		Healthy *n* = 24 (PBMC)			
		Asthmatics *n* = 30 (PBMC)			

**TABLE 3:** Emerging role of circular RNAs in COPD.

Name of the CircRNA	Expression level	Key role	Mechanisms	References
Circ-HACE1	↑ in the lung of smokers without or with COPD and 16HBE cells exposed to CSE	Promotes CSE-induced 16HBE cells injury	By modulating TLR4 by Sponging miR-485-3p	Zhou et al. (2021a)
CircFOXO3	↑ in CS-exposed lungs and CSE-exposed murine alveolar epithelial cells	Silencing ameliorates inflammatory response in the lungs of CS-exposed mice	CircFOXO3/miR-214-3p/NF-κB signaling pathway	Zhou et al. (2021b)
CircBbs9	↑ in the lung of PM_2.5_ exposed and COPD model in mice	Promotes PM_2.5_-induced lung inflammation in mice	Through activation of NLRP3 inflammasome via regulating miR-30e-5p-Adar	Li et al. (2020)
Circ_0008672	↑ in PBMCs of COPD patients	targetes NOD-like receptor signaling pathway, natural killer cell-mediated cytotoxicity, and Th17 cell differentiation	circ_0008672/miR-1265/MAPK1 signaling axis in COPD	Duan et al. (2020)
circ-BPTF	↑ in the pulmonary arteries of COPD patients	Promotes hypoxic PASMC proliferation	Circ-BPTF/miR-486-5p/CEMIP axis	Wang et al. (2022b)
Circ_0006892	↓ in the lung of smokers with COPD and 16HBE cells exposed to CSE	Regulates 16HBE cells apoptosis and inflammation	Via modulating miR-24/PHLPP2 axis	Zhang et al. (2022)
Circ_OSBPL2	↑ in the lung of smokers with or without COPD and CSE-treated HBECs	facilitates apoptosis, inflammation, and oxidative stress	Via miR-193a-5p/BRD4 axis	Zheng et al. (2021)
	↑ in the serum of smokers with and without COPD	Promotes 16HBE cells dysfunction	By regulating miR-515-5p/IGFBP3 axis	Miao et al. (2022)
CircANKRD11	↑ in the lung tissues of smokers with or without COPD and HPMECs exposed to CSE	knockdown attenuates HPMECs apoptosis, inflammation, and oxidative stress	by regulating miR-145-5p/BRD4 Axis	Wang et al. (2021b)
Circ_0006872	↑ in the lung of smokers with and without COPD	Promotes apoptosis, inflammation, and oxidative stress in HPMECs and BEAS-2B cells	By modulating miR-145-5p/NF-κB pathway	Xue et al. (2021)
Circ_0061052	↑ in CSE-treated 16HBE	Regulates airway remodeling and EMT	Circ_0061052/miR-515-5p/FoxC1/Snail axis	Ma et al. (2020)
Circ-RBMS1	↑ in COPD patients and 16HBECs exposed to CSE	Silencing attenuates apoptosis, inflammation, and oxidative stress	Circ-RBMS1/miR-197-3p/FBXO11 axis	Qiao et al. (2021)
circXPO1	↑ in the lungs of CS-exposed mice and CSE exposed AT2 cell line	Inhibition attenuates inflammation and cellular senescence	CircXPO1/miR-23b-3p/TAB3 signaling axis	Du et al. (2022)
Circ_0026466	↑ in the blood of smokers with COPD and CSE-induced HBECs	Knockdown attenuates CSE-induced inhibition of the viability and proliferation of HBECs and induced cell apoptosis, inflammation, and oxidative stress	Circ_0026466/miR-153-3p/TRAF6/NF-κB pathway	Wang et al. (2023b)

**TABLE 4:** Biomarker role of circRNAs in COPD.

CircRNAs	Expression level	Study size	Biomarker role	Biomarker type	Refs
Circ_0001859	↓ in serum of COPD and AE-COPD patients	Healthy *n* = 28	Higher specificity and sensitivity in predicting COPD and AE-COPD, and correlated with FEV1% predicted	Diagnostic and prognostic	Chen et al. (2020)
		COPD *n* = 38			
		AE-COPD *n* = 24			
Circ_0040929	↑ in the blood of smokers with and without COPD	Non-smokers *n* = 22	Circ_0040929 expression was higher in COPD compared to smokers and non-smokers	Diagnosis	Miao et al. (2022)
		Smokers *n* = 22			
		COPD *n* = 22			

## CircRNAs and asthma

### CircRNAs and airway smooth muscle cells (ASMCs)

Although mild asthma is effectively manageable, severe asthma remains a substantial source of economic and social burden. It often involves substantial changes in ASMCs, such as hyperplasia, hypertrophy, and alterations in inflammation and fibrosis-related proteins (Camoretti-Mercado and Lockey, 2021). These aberrant ASMCs greatly influenced airway remodeling in severe asthma (Hershenson et al., 2008). Unfortunately, there are limited interventions to address these ASMC abnormalities. Thus, a solid understating the pathogenesis of asthma is crucial for developing effective therapeutic strategies to alleviate the impact of severe asthma on ASMCs and improve patient outcomes.

Recent advancements in next-generation sequencing technology have facilitated the identification of numerous circRNAs across a wide range of cell types and species (Kristensen et al., 2019). However, limited research has explored the involvement of circRNAs in ASMCs dysfunction in asthma. Over the past few years, a growing body of research studies has uncovered circRNAs as regulators of various cellular and biological processes in asthmatic ASMCs, including proliferation, apoptosis, migration, ECM production, and secretion of inflammatory mediators, primarily through acting as ceRNAs of miRNAs to regulate target gene mRNAs.

One such circRNA is circERBB2, derived from the ERBB2 gene locus, associated with tumorigenesis. Elevated circERBB2 expression was observed in bronchial biopsy tissues of asthmatic individuals and in Platelet-Derived Growth Factor BB (PDGF-BB)-stimulated ASMCs, an *in vitro* model of asthma (Huang J. Q. et al., 2021). PDGF-BB is a well-known factor that stimulates airway remodeling and inflammation in asthma (Kardas et al., 2020). It promotes proliferation and migration of ASMCs (Ito et al., 2009; Spinelli et al., 2012). It is also crucial to highlight that the upregulation of PDGF-BB expression is closely associated with the severity of asthma, and increased proliferation and migration of ASMCs are commonly observed in individuals with asthma (Ohno et al., 1995). Knockdown of circERBB2 using small hairpin RNA (shRNA) led to significant amelioration of inflammation, proliferation, and migration in ASMCs induced by PDGF-BB (Huang J. Q. et al., 2021). Mechanistically, circERBB2 functions as a sponge for miR-98-5p, modulating IGF1R expression and accelerating ASMC proliferation and migration upon PDGF-BB treatment (Huang J. Q. et al., 2021).

Another circRNA, circHIPK3, also known as circ_0000284, was upregulated in ASMCs when stimulated with PDGF-BB (Lin et al., 2020; Jiang et al., 2021). CircHIPK3 was found to promote migration and proliferation while suppressing apoptosis in PDGF-BB-stimulated ASMCs. It achieved this through interacting with miR-326 to regulate stromal interaction molecule 1 (STIM1) (Lin et al., 2020), an endoplasmic reticulum membrane protein known that acts as a calcium sensor and stimulator of ASMCs remodeling and AHR in asthma (Zou et al., 1985; Johnson et al., 2022). Moreover, circHIPK3 was shown to boost ASMC proliferation, invasion, and migration upon PDGF treatment, potentially through the regulation of the miR-375/matrix metalloproteinase-16 (MMP-16) axis (Jiang et al., 2021).

PDGF-treated ASMCs also exhibited upregulation of circ_CSNK1E and circ_0002594, while the expression of circ_0000029 was downregulated (Ding et al., 2022; Quan et al., 2022; Wang R. et al., 2023). Furthermore, the expression of circ_0002594 and circ_CSNK1E was significantly increased in samples obtained from patients with asthma (Ding et al., 2022; Quan et al., 2022). Functional studies demonstrated that the shRNA-mediated knockdown of circ_CSNK1E significantly reduced the proliferative and migratory responses of ASMCs to PDGF (Ding et al., 2022). Computation analysis and experimental validation studies revealed that circ_CSNK1E binds to miR-34a-5p to modulate vesicle-associated membrane protein 2, thereby accelerating PDGF-induced ASMCs proliferation and migration (Ding et al., 2022). Moreover, circ_0002594 was identified as a facilitator of inflammation, migration, and proliferation in ASMCs in response to PDGF treatment. The authors further demonstrated that circ_0002594 acts as a sponge for miR-139-5p to modulate the expression of tripartite motif 8 (TRIM8), which in turn stimulates cellular damage (Quan et al., 2022). Studies have also shown that miR-139-5p treatment and TRIM8 inhibition decreases ASMCs proliferation through inhibiting a chromatin remodeling factor BRG1, and inactivating NF-kb signaling pathway, respectively (Zhang et al., 2017; Dang et al., 2020). Another circRNA, circ_0000029 was found to regulate ASMC migration and proliferation by modulating miR-576-5p and KCNA1 expression (Wang R. et al., 2023). These findings underscore the substantial role of circRNAs in modulating critical functions in asthmatic ASMCs through their interactions with microRNAs and target genes. However, the full extent of circRNA functions with other mechanisms in asthmatic ASMCs remains largely unexplored. Further research is required to gain a comprehensive understanding of the role played by circRNAs in asthmatic ASMCs.

### CircRNAs and airway and bronchial epithelial cells

Airway epithelial cells serve as the initial protective barrier, guarding the airway and lungs against inflammatory stimuli and antigens. Dysfunction of airway and bronchial epithelial cells is commonly found in all types of asthma. Emerging evidence indicates that aberrant expression of circRNAs may exert a substantial influence on the functionality of airway and bronchial epithelial cells (Table 1).

Jia et al. conducted a comprehensive profiling study of circRNAs using micro-arrays in human bronchial epithelial cells (BEAS-2B) exposed to PM2.5, a known respiratory hazard associated with lung diseases, including asthma (Jia et al., 2020). Among the dysregulated circRNAs, circ_406961 showed a remarkable fold change difference. Experimental investigations revealed a dose-dependent decrease in circ_406961 expression upon PM2.5 exposure in BEAS-2B cells. Silencing circ_406961 using siRNA reduced cell viability and increased inflammation, while its overexpression reversed these effects. Mechanistic analysis highlighted the role of circ_406961 in modulating inflammation by interacting with ILF2 and activating the STAT3/JNK pathways.

Another circRNA circVPS33A was shown to be upregulated in the plasma of asthmatics and BEAS-2B cells treated with house dust mites (HDM) protein (Der p1) (Su et al., 2021). Der p1 treatment resulted in decreased cell viability, proliferation, migration, invasion, and induced apoptosis, autophagy, and inflammation in BEAS-2B cells. Silencing circVPS33A using siRNA ameliorated these cellular damages induced by Der p1. Mechanistically, circVPS33A was shown to promote these cellular damages by acting as a sponge for miR-192-5p, leading to modulating the expression of HMGB1. Furthermore, circARRDC3 was found to enhance inflammation and mucus production in nasal epithelial cells stimulated with IL-13 in allergic rhinitis, a substantial risk factor for the development of asthma (Wang T. et al., 2021). This regulatory effect was mediated through the miR-375/KLF4 signaling axis. Together, these findings suggest that dysregulated circRNAs may contribute to bronchial epithelial cell dysfunction associated with asthma. However, these findings are needed to further confirm in *in vivo* animal models and clinical studies.

### CircRNAs and airway goblet cells

Airway goblet cells play a vital role in maintaining airway homeostasis through the production and secretion of mucins (Whitsett, 2018). In response to genetic factors, growth factors, inflammation or environmental insults, goblet cells differentiate from club (Clara) cells, a type of airway epithelial cells (Whitsett, 2018; Kim et al., 2019). Uncontrolled differentiation of the goblet cells leads to increased production and secretion of mucins within the airways (Fahy and Dickey, 2010). Excessive accumulation of mucins is a common manifestation of many airway diseases, such as asthma, COPD, and cystic fibrosis (Fahy and Dickey, 2010). In asthma, goblet cell hyperplasia (increased number of goblet cells) is one of the major features of airway remodeling and is associated with chronic mucus hypersecretion and resultant airway obstruction (Rogers, 2002). Recently, circRNAs have been shown to induce goblet cell hyperplasia. For example, Wang et al., discovered that circZNF652, also known as circ_0000782, can promote goblet cell hyperplasia in allergic epithelial (Wang X. et al., 2022). CircZNF652 exhibited significant and specific expression in the airway epithelium of both children with asthma and ova-induced mice models of experimental asthma (Wang X. et al., 2022). The increased expression of circZNF652 was found to contribute to the promotion of bronchial goblet cell metaplasia and the excessive secretion of mucus. It was discovered that circZNF652 accelerated goblet cell metaplasia through sponging miR-452-5p and activating JAK2/STAT6 signaling axis (Wang X. et al., 2022). Furthermore, the splicing factor ESRP1 was identified as a facilitator of circZNF652 biogenesis, thereby accelerating goblet cell metaplasia (Wang X. et al., 2022). These findings highlight the significant role of the ESRP1/circZNF652/miR-452-5p/JAK2/STAT6 axis in the modulation of goblet cell metaplasia, mucus hypersecretion, and AHR in mice models of experimental asthma. Therefore, targeting circZNF652 or activating miR-452-5p could offer a potential therapeutic strategy for intervening in epithelial remodeling in experimental asthma. Nevertheless, the exact underlying mechanism responsible for the elevation of ESRP1 expression in allergic airway epitheliums is yet unclear. Furthermore, circZNF652 has been identified as a regulator of EIF5, RhoA, JAK2, and SRSF3. While the involvement of JAK2 in goblet cell metaplasia in asthma has been established, the roles of other factors in this process are still unclear and require further investigation.

### CircRNAs and immune cells

Asthma involves the infiltration and activation of various immune cells in the airways. These immune cells encompass dendritic cells (DCs), T cells (Th1, Th2, Th9, Th17, Th22 cells), innate lymphoid cells (ILCs), eosinophils, neutrophils, B cells, and mast cells (Lommatzsch, 2020). Asthma can be categorized into two primary types: type 2 (the most common) and non-type 2 asthma. Type 2 asthma is typically triggered by allergens but can also be provoked by non-allergic factors. In contrast, non-type 2 asthma is primarily induced by non-allergic factors such as pollutants and infections. Upon recognizing allergens, Th2 cells or ILCs, especially ILC2, release cytokines like IL-4, IL-5, and IL-13, leading to the recruitment of eosinophils, which cause airway inflammation (Lommatzsch, 2020). Mast cells release histamine, contributing to bronchoconstriction. Neutrophils may also be involved in severe asthma. Dendritic cells present antigens to T cells, initiating immune responses. Macrophages can promote either inflammation or resolution depending on the types of macrophages (M1 or M2). B cells produce IgE antibodies, which trigger allergic responses. These immune mechanisms collectively lead to the characteristic symptoms of asthma, such as bronchoconstriction and AHR.

Emerging studies have provided substantial evidence supporting the pivotal role of circRNAs as competing endogenous RNAs (ceRNAs) for microRNAs in T cell development and function. An example of circRNA involvement in immune regulation is the LPS-induced circRNA, m_circRasGEF1B, which regulates the stability of ICAM-1 mRNA, contributing to immune regulation in the body (Ng et al., 2016). ICAM1, a cell surface glycoprotein, is expressed in endothelial cells and specific immune cells and is well-recognized as a biomarker for inflammation. Analysis of circRNA expression profiles in different cell subpopulations has been demonstrated to possess distinct expression profiles, indicating their regulatory roles in specific cellular processes. For example, studies have revealed that the decreased hsa_circ_0012919 expression leads to CD11a and CD70 DNA methylation in CD4^+^ T cells (Wang et al., 2015; Li L. J. et al., 2018; Zhang et al., 2018; Gaffo et al., 2019). Furthermore, hsa_circ_0045272 has been demonstrated to potentially act as a negative regulator of T cell apoptosis promotion and IL2 release by interacting with hsa-miR-6127 (Li L. J. et al., 2018). Moreover, circTNIK, circIKZF1, circFBXW7, and circTXK are circRNAs specifically expressed in T cells (Gaffo et al., 2019).

In recent years, a wealth of evidence has emphasized the substantial role of circRNAs in the pathophysiology of asthma through modulating immune responses (Ghafouri-Fard et al., 2020; Chen et al., 2021). Substantial evidence underscores the essential role of CD4^+^ T cells in driving the disease by regulating IgE production in response to allergic conditions, secreting pro-inflammatory mediators, and activating and recruiting inflammatory cells like eosinophils, macrophages, and neutrophils (DiCosmo et al., 1994; Erpenbeck et al., 2003; Ferreira, 2003; Fujiwara et al., 2007; Holgate, 2008; Lloyd and Hessel, 2010; Fujimoto et al., 2011; Lambrecht and Hammad, 2015; Chen et al., 2016; Cheng et al., 2016; Hammad and Lambrecht, 2021). CD4^+^ T cells exhibit notable cytokine production abilities, with Th1 cells producing IFN-γ, Th2 cells secreting IL-4, IL-5, IL-6, IL-9, and IL-13, and Th17 cells releasing IL-17A and IL-17F (Lambrecht and Hammad, 2015). The complex immune response observed in asthma is regulated by this intricate network of diverse cytokines (Erpenbeck et al., 2003; Zhu and Paul, 2010; Fujimoto et al., 2011; Chen et al., 2016; Cheng et al., 2016). To explore the critical involvement of circRNAs in CD4^+^ T cells in asthma, Huan et al. profiled the expression of circRNAs using micro-array analysis in CD4^+^ T cells isolated from 5 asthmatics and 5 healthy subjects (Huang et al., 2019). This study revealed 597 dysregulated circRNAs in asthmatic patients compared to the control group. Among the dysregulated circRNAs, hsa_circ_0005519 exhibited one of the highest fold of up-regulations, which was further validated through subsequent qRT-PCR analysis in the existing cohort samples as well as in a separate cohort comprising 65 asthma patients and 30 healthy subjects. The authors employed bioinformatic analysis, reporter assays, and literature search to uncover that hsa_circ_0005519 functions as a regulator of the expression of IL13 and IL6, possibly through interacting with let-7a-5p. Notably, IL-13 and IL-6 have been widely recognized for their significant involvement in asthma pathogenesis (DiCosmo et al., 1994; Zhu et al., 1999; Pope et al., 2001; Kimura and Kishimoto, 2010). Furthermore, the expression of hsa_circ_0005519 in CD4^+^ T cells was found to exhibit a negative correlation with let-7a-5p, and a positive correlation with IL13 and IL6 mRNA, as well as FeNO and peripheral blood eosinophil ratio. These results indicate that hsa_circ_0005519 may be a promising biomarker for asthma.

In a separate study, the authors identified another circRNA, hsa_circ_0002594, which exhibited upregulation in CD4^+^ T cells using the existing micro-array dataset consisting of 5 patients with asthma and 5 healthy controls (Huang Z. et al., 2021). To validate the findings, a second cohort consisting of 83 asthmatics and 54 healthy subjects was examined, and the result revealed a significant increase in hsa_circ_0002594 levels in CD4^+^ T cells of asthmatics (Huang Z. et al., 2021). In asthmatics, the expression of hsa_circ_0002594 in CD4^+^ T cells showed a positive correlation with FeNO and a negative association with PD20 (the methacholine dose required to induce a 20% decrease in FEV1). The authors performed a correlation analysis of hsa_circ_0002594 expression in the subgroups of asthmatic patients with clinical features. The researchers observed that the subgroup with high expression of hsa_circ_0002594 exhibited elevated FeNO levels and lower PD20 values compared to the subgroup with low expression of hsa_circ_0002594. Furthermore, the subgroup with high expression of hsa_circ_0002594 had higher Th2-high and SPT-positive individuals, when compared to non-Th2 inflammation and SPT-negative cases. These findings suggest that the subgroup of asthmatics with high expression of hsa_circ_0002594 also exhibits clinical indicators consistent with Th2-driven allergic response. The treatment with inhaled ICS led to a significant reduction in hsa_circ_0002594 expression, highlighting its potential as a therapeutic target. Moreover, in exploring the diagnostic potential of hsa_circ_0002594 for asthma, the authors observed that its expression demonstrated high sensitivity and specificity values in diagnosing asthma, regardless of the presence or absence of ICS treatment. Furthermore, the authors investigated the potential molecular mechanisms of hsa_circ_0002594 in the development of asthma through computational analysis and identified potential target miRNAs of the circRNA. They proposed the hsa_circ_0002594 could competitively sequester the activity of hsa-let-7e-5p, hsa-miR-503-5p, hsa-miR-16-5p, hsa-miR-587, and hsa-miR-514a-3p. Although this study had several limitations: 1) Small sample size necessitates larger, diverse studies. 2) MicroRNA interactions with hsa_circ_0002594 lack experimental validation. 3) Circulating hsa_circ_0002594 levels were not assessed for biomarker potential.

In a recent study, it was discovered that mmu_circ_0001359 exhibited downregulation in the lungs of an OVA-induced murine asthma model (Shang et al., 2020). Moreover, the delivery of exosomes derived from ADSCs, which were modified with mmu_circ_0001359, was observed to mitigate airway remodeling by facilitating the activation of M2-like macrophages through the regulation of the miR-183-5p/FoxO1 signaling pathway (Shang et al., 2020).

In another recent study, circRNAs were profiled in the lung of mice with experimental asthma developed by HDM (Bao et al., 2020). Through the analysis of the circRNA-miRNA signaling axis, two upregulated circRNAs, circ_0000629 and circ_0000455, were identified to target miR-29b and miR-15a, respectively. Earlier investigations have demonstrated an inverse association between these miRNAs and the occurrence of allergic reactions (Nakano et al., 2013; Yan et al., 2019). In contrast, two downregulated circRNAs, circ_0000723 and circ_0001454 were found to target miR-214 and miR-146b respectively. MiR-214 and miR-146b have previously been positively associated with asthma (Feng et al., 2012; Qiu et al., 2017). Thus, these four circRNAs represent promising candidates for further investigation concerning their association with asthma.

### CircRNAs in COPD

Increasing evidence suggests that circRNAs are implicated in the pathophysiology of COPD, as shown in Tables 3, 4 and Figure 3. To identify circRNAs involved in COPD, several circRNAs profile studies have been profiled using NGS technology. As an example, Duan et al., profiled circRNA expression in PBMCs of 21 patients with COPD and 21 healthy individuals (Duan et al., 2020). The analysis revealed 2,132 differentially expressed circRNAs and 2,734 differentially expressed mRNAs in COPD patients. The further in-depth analysis identified an association between circ_0008672 and key COPD pathways, including Th17 cell differentiation, NK cell-driven cytotoxicity, and NOD-like receptor pathway. The authors also constructed a circRNA-microRNA-mRNA network to investigate circRNAs’ role as microRNA sponges in COPD. Their findings showed that circRNAs can regulate mRNAs by acting as sponges for one or multiple miRNAs. For instance, circ_0008672 was discovered to interact with miR-1265 and thereby regulate MAPK1 expression. Notably, MAPK signaling activation plays a crucial role in the development of various pathophysiological features of COPD, including, but not limited to, lung inflammation, airway mucus hypersecretion, airway fibrosis, and T cell activation (Mercer and D'Armiento, 2006). These findings underscore the ceRNA network’s importance in COPD, but limitations exist. Validation in a larger COPD cohort is essential. Experimental studies on circRNA functions are needed for precise roles and also subsets of PBMCs warrant assessment for comprehensive insights.

Circ-HACE1, also known as circ_0077520, was over-expressed in the serum of 21 smokers and 24 smokers with COPD compared to 17 non-smokers (Zhou F. et al., 2021). Additionally, in human bronchial epithelial cells (16HBE cells) exposed to cigarette smoke extract (CSE), a well-established *in vitro* model of COPD, a significant increase in the expression of circ-HACE1 was observed (Zeng et al., 2019; Zhou F. et al., 2021). Targeting circ-HACE1 expression using siRNA demonstrated an improvement in CSE-induced cellular damage in 16HBE cells, as evidenced by increased cell viability and reduced inflammation, oxidative stress, and apoptosis. This beneficial effect was achieved through circ-HACE1’s interaction with miR-485-3p, which modulated the expression level of TLR4. TLR4 plays a crucial role in innate immune activation and is closely linked to inflammatory responses in several conditions, including diabetes. These findings highlight circ-HACE1 as a promising target to enhance the diagnostic accuracy of COPD and suggest that inhibiting its expression could hold potential benefits in the therapeutic management of COPD.

Furthermore, circ_0040929 showed significant upregulation in both serum of patients with COPD and 16HBE cells exposed to CSE (Miao et al., 2022). Circ_0040929 silencing protected from CSE-treated 16HBE cell injuries via the regulation of the miR-515-5p/IGFBP3 signaling axis. Circ-RBMS1, also known as has_circ_0002136, originates from the RBMS1 gene and was shown to be over-expressed in the PBMCs of patients with COPD (Duan et al., 2020). However, the specific role and molecular mechanisms of circ-RBMS1 were not further investigated in this circRNA profiling and bioinformatic analysis study. Recently, Qiao et al. also found upregulation of circ-RBMS1 in both patients with COPD (Qiao et al., 2021). Furthermore, circ-RBMS1 expression was shown to increase in a dose-dependent manner in CSE-treated 16HBE cells (Qiao et al., 2021). Functional analysis demonstrated that inhibiting circ-RBMS1 with siRNA resulted in the attenuation of CSE-induced 16HBE cell injuries. Mechanistically, circ-RBMS1 was found to interact with miR-197-3p to modulate FBXO11, a member of the F Box family of proteins known for its role in ubiquitination and degradation of substrates, as well as genome stability regulation. A previous study also associated FBXO11 with airway remodeling, apoptosis, and inflammation induced by CSE (Mei et al., 2020). However, it is important to consider that these findings were obtained from *in vitro* analysis. To validate and strengthen these results, further investigation using *in vivo* assays and a larger COPD patient cohort will be necessary.

CircFOXO3 was increased in mouse alveolar epithelial cells exposed to CSE and in the lung of mice with experimental COPD induced by CS (Zhou L. et al., 2021). However, silencing CircFOXO3 using lentivirus attenuated CS-induced lung inflammation in mice. This effect was mediated through the CircFOXO3/miR-214-3p/NF-κB signaling pathway, targeting the inflammatory response induced by CS in the lungs of mice. Thus, targeting circFOXO3 could serve as a novel preventive approach to counter CS-induced inflammation.

The Circ-OSBPL2 was found to regulate apoptosis, oxidative stress, and inflammation in HBECs exposed to CSE by modulating the miR-193a-5p/BRD4 axis (Zheng et al., 2021). Circ_0061052 was upregulated in CSE-exposed HBECs and modulated airway remodeling and EMT induced by CS through sponging miR-515-5p, thereby regulating downstream targets FoxC1 and Snail (Ma et al., 2020). Circ_0026466 exhibited upregulation in the blood of smokers with COPD and CSE-exposed HBECs (Wang C. et al., 2023). Knockdown of circ_0026466 mitigated CSE-induced inhibition of viability and proliferation, induced apoptosis, oxidative stress, and inflammation in HBECs through the circ_0026466/miR-153-3p/TRAF6/NF-κB pathway (Wang C. et al., 2023). Similarly, circXPO1 displayed upregulation in the lung of mice with experimental COPD induced by CS and CSE-treated AT2 cell lines (Du et al., 2022). Inhibition of circXPO1 was found to suppress inflammatory response and cellular senescence induced by CSE. Mechanistic experiments revealed that circXPO1 regulates TAB3 mRNA through sponging miR-23b-3p to modulate the inflammatory response and cellular senescence processes. The findings suggest that circXPO1 contributes to COPD pathogenesis potentially through sponging miR-23b-3p, thereby modulating TAB3 expression.

In contrast, the downregulation of circRNAs can also contribute to COPD pathogenesis. For instance, circ_0006892 was found to be downregulated in the lungs of COPD patients and CSE- 16HBE and BEAS-2B cells exposed to CSE, as measured by qRT-PCR (Zhang et al., 2022). Furthermore, the expression of circ_0006892 positively correlated with FEV1% in COPD patients. To explore the impact of circ_0006892, gain-of-function experiments were conducted on 16HBE and BEAS-2B cells exposed to CSE, assessing cell survival, apoptosis, and inflammation. The results indicated that over-expression of circ_0006892 through plasmid transfection mitigated cell apoptosis and the inflammatory response induced by CSE, indicating its protective function in CSE-associated bronchial epithelial injury. Conversely, inhibition of circ_0006892 led to increased apoptosis and inflammation in HBECs exposed to CSE, and this effect was regulated through the modulation of the miR-24/PHLPP2 axis. However, it is crucial to consider that because this study only examined the *in vitro* performance of circ_0006892, its conclusions might not accurately reflect the complicated environment experienced by COPD patients. To gain a comprehensive understanding of COPD pathogenesis, further investigations using preclinical animal models are warranted.

CircRNAs have also been shown to play a regulatory role in pulmonary endothelial and smooth muscle cells in the context of COPD *in vitro* models. For example, circANKRD11 demonstrated upregulation in the lungs of smokers with or without COPD and CSE-treated human pulmonary microvascular endothelial cells (HPMECs) (Wang Z. et al., 2021). Knocking down circANKRD11 in HPMECs resulted in a reduction of cellular apoptosis, inflammatory response, and oxidative stress induced by CSE, achieved through the modulation of the miR-145-5p/BRD4 axis. Elevated expression of Circ-BPTF was found to be over-expressed in the pulmonary arteries of patients with COPD, and it facilitated the proliferation of hypoxic pulmonary artery smooth muscle cells (PASMCs). Circ-BPTF/miR-486-5p/CEMIP signaling axis promoted the proliferation of the PASMCs (Wang C. et al., 2022). While dysfunction of pulmonary vascular smooth muscle cells and endothelial cells is well-established in pulmonary arterial hypertension, their involvement as major contributors to chronic vascular inflammation and lung damage in COPD is increasingly recognized. Future studies are warranted to explore the causal function of circRNAs in these cells through *in vivo* and clinical investigations.

Overall, dysregulation of circRNAs is evident in COPD cells, and they play significant roles in disease development and progression. However, it is important to note that these findings primarily originate from *in vitro* experiments and may not fully represent the complex environment of COPD patients. To gain a better understanding of COPD pathogenesis and further investigate the role of circRNAs, future studies utilizing pre-clinical animal models are warranted.

### CircRNAs as potential biomarkers in asthma and COPD

As mentioned above, circRNAs exhibit stability due to their closed-loop structures, conservation across species, and display tissue- and developmental stage-specific expression manners. Moreover, circRNAs are abundantly present in blood, saliva, exosomes, and urine, enabling their detection with remarkable sensitivity and specificity. These distinctive features make circRNAs promising candidates for biomarker discovery.

CircRNAs have emerged as potential biomarkers in asthma and COPD. For instance, receiver operating characteristic (ROC) curve analysis of circ_0002594 expression in the CD4^+^ T cells of 83 asthmatics who were not using inhaled corticosteroids (ICS) revealed a significantly high area under the curve (AUC) value of 0.727 (Huang Z. et al., 2021). Similarly, an AUC value of 0.767 were found when assessing the diagnostic utility of circ_0002594 in the context of ICS treatment in 48 asthmatic patients (Huang Z. et al., 2021). Furthermore, the expression of circ_0002594 showed a positive correlation with exhaled nitric oxide levels, the patient’s family history, positive skin prick test (SPT) results, and Th2 cytokine expression levels (Huang Z. et al., 2021). Additionally, elevated expression of circSORT1 and circSERPINB1 was observed in the induced sputum of 68 asthmatic patients compared to 20 healthy individuals, demonstrating higher specificity and sensitivity in predicting asthma (Xu et al., 2023). CircSORT1 expression correlated with various asthma clinical parameters, including FeNO, EOS%, IL-17A, IFN-γ, and PC20 (Xu et al., 2023). On the other hand, CircSERPINB1 expression showed correlations with IL-6, IL-17A, IFN-γ, FEV1%, and FVC% (Xu et al., 2023). Furthermore, a significantly increased expression of circ_0005519 was observed in PBMCs and CD4^+^ T cells of 30 asthmatics compared to 24 healthy individuals, and this increased expression was associated with exhaled nitric oxide levels (Huang et al., 2019). The serum levels of circ_0001859 were observed to be reduced in 38 patients with COPD and 24 patients with acute exacerbation of COPD (AE-COPD) compared to 28 healthy controls. This decreased expression exhibited higher specificity and sensitivity in predicting both COPD and AE-COPD and correlated with the FEV1% predicted. Furthermore, another circRNA, circ_004092 expression was found to be increased in the blood of smokers with COPD (*n* = 22) or smokers without COPD patients (*n* = 22) in comparison to non-smokers (*n* = 22). Collectively, these findings indicate that circRNAs hold promise as potential diagnostic and prognostic biomarkers for asthma and COPD. However, the potential of circRNAs as biomarkers in asthma and COPD requires validation in larger sample cohorts.

## Conclusion and future perspectives

Circular RNAs (circRNAs) show promise in asthma and COPD research, offering potential as therapeutic targets and biomarkers. Despite valuable insights gained, significant challenges remain. Limited circRNAs have been linked to asthma and COPD, underscoring the need for a more comprehensive circRNA profile in these conditions. These molecules play crucial roles in various airway and immune cell functions, including AECs, 16HBE, ASMCs, CD4^+^ T cells, and macrophages in asthma, and 16HBE and AECs in COPD. However, no research has delved into circRNA roles in ASMCs dysfunction in COPD, despite its pivotal role in airway remodeling. Further investigations are necessary to enhance our understanding of COPD. Moreover, the primary focus of asthma and COPD studies has been on circRNAs’ capacity to sponge microRNAs, leaving other regulatory mechanisms such as protein sponges and scaffolds. Additionally, while most circRNA studies have been confined to *in vitro* settings, their *in vivo* roles remain mostly unexplored. Only a select few, such as circZNF652 in asthma, and circFOXO3, circFOXOP1, and circBbs9 in COPD, have been studied in animal models, with no assessment of their impact on lung function changes in asthma and COPD. Exploring the clinical applicability of circRNAs as diagnostic tools, prognostic markers, and predictive biomarkers requires studies in extensive patient cohorts. Finally, circRNAs offer potential for targeted therapeutic approaches, demanding identification of circRNAs with therapeutic value and the development of strategies to modulate their expression or activity, potentially leading to innovative asthma and COPD treatments.
